# Mental Health Consequences of Lockdown During the COVID-19 Pandemic: A Cross-Sectional Study

**DOI:** 10.3389/fpsyg.2021.605279

**Published:** 2021-02-26

**Authors:** Ahmed Msherghi, Ali Alsuyihili, Ahmed Alsoufi, Aimen Ashini, Zenib Alkshik, Entisar Alshareea, Hanadi Idheiraj, Taha Nagib, Munera Abusriwel, Nada Mustafa, Fatima Mohammed, Ayah Eshbeel, Abobaker Elbarouni, Muhammed Elhadi

**Affiliations:** ^1^Faculty of Medicine, University of Tripoli, Tripoli, Libya; ^2^Faculty of Medicine, Sabha University, Sabha, Libya; ^3^Faculty of Medicine, University of Zawia, Zawia, Libya; ^4^Faculty of Medicine, University of Misrata, Misrata, Libya

**Keywords:** COVID-19, SARS-CoV-2, lockdown, psychology, mental health, anxiety, stress

## Abstract

**Objective:**

We aimed to provide an overview of the psychological status and behavioral consequences of the lockdown during the COVID-19 pandemic in Libya.

**Methods:**

A cross-sectional study was conducted among the Libyan population through May and June 2020 in more than 20 cities. The survey comprised basic demographic data of the participants and anxiety symptoms measured using the seven-item Generalized Anxiety Disorder scale (GAD-7) with ≥15 as the cut-off score for clinically significant anxiety symptoms. Additionally, a survey regarding the lockdown effect was administered, which consisted of several parts, to measure the lockdown status.

**Results:**

A total of 8084 responses were recorded, of which, 5090 (63%) were women and 2994 (37%) were men. The mean age (SD) for study participants was 27.2 (8.9) years. Among the participants, 1145 (14.2%) reached the cut-off score to detect anxiety symptoms; however, of the study variables, only five were predictors of clinically significant anxiety: age, gender, marital status, work status, being a financial supporter for the family, and being infected with COVID-19. Women had 1.19 times higher odds to exhibit anxiety symptoms than men. Increasing age was significantly associated with reduced likelihood of exhibiting anxiety symptoms, whereas being married was significantly associated with higher likelihood of anxiety symptoms, compared to not being married. Being suspended from work was associated with an increase in the likelihood of anxiety symptoms. However, we found that being infected with COVID-19 was associated with a 9.59 times higher risk of exhibiting severe anxiety symptoms. Among the study participants, 1451 (17.9%) reported a physical and/or verbal abuse episode from family members, 958 (11.9%) reported abuse outside the family, and 641 (7.9%) reported abuse from enforcers, during the lockdown.

**Conclusion:**

Our study provided an overview of the psychological and behavioral status, among those who resided in Libya during the civil war and COVID-19 pandemic. The study demonstrates a concerningly high level of clinically significant anxiety during lockdown among the Libyan population during Libya’s lockdown period.

## Introduction

Severe acute respiratory syndrome coronavirus-2 was identified as the cause of coronavirus disease 2019 (COVID-19). Since then, this infectious disease has caused unprecedented challenges for healthcare systems around the world, resulting in a pandemic that has resulted in greater than 96.2 million cases of COVID-19 and greater than 2.06 million deaths worldwide, as of January 21, 2021 (Dong et al., 2020).

The COVID-19 pandemic has affected the population and societies around the world, raising anxiety, stress, and an increased financial burden effect of unemployment and resulting in people losing their jobs, which has had negative consequences on families and individuals’ behaviors and perceptions of life (Taylor, 2019; Gualano et al., 2020; Ni et al., 2020).

As the pandemic has spread worldwide, authorities and societies have imposed administrative restrictive measures and lockdown procedures to prevent the spread of the viral infection (Alfano and Ercolano, 2020; Lau et al., 2020; Moris and Schizas, 2020). Libya has implemented several restrictive procedures to control virus spread since March 2020, including lockdown measures.

These measures can have a dramatic effect on the mental health of people. The lockdown adds to the effect endured by people who lost their job due to the pandemic, which results in higher pressure on families to generate adequate financial support. The isolation and lockdown measures have several negative mental health consequences, which are due to family separation, loss of personal freedom, and the stress related to socioeconomic consequences of the pandemic (Yao et al., 2020). During a global pandemic, people in special lockdown settings are in need of medications, food, and an ability to maintain a normal lifestyle, therefore these concerns should be addressed (Matias et al., 2020). Further, the sleep and weight changes that have occurred during the lockdown period should be addressed (Huang and Zhao, 2020; Sher, 2020).

A study conducted among 4872 participants in 31 provinces and regions in China found that 22.6% have anxiety symptoms, and 48.3% have depressive symptoms (Gao et al., 2020). Another study conducted among 3480 participants from Spain found that 21.6% displayed anxiety symptoms, and 18.7% have depressive symptoms (González-Sanguino et al., 2020). A similar study in Italy, found 18.7% have anxiety symptoms (Mazza et al., 2020). Another study conducted in Turkey among 343 participants in the young age group revealed high anxiety symptoms in 45.1% (Özdin and Bayrak Özdin, 2020). However, there was no recent study among the African general population, as most of the recently published studies were in other regions and continental areas other than Africa (Xiong et al., 2020). Therefore, there is a need to assess the consequences of COVID-19 lockdown on the African population’s behavioral and mental status.

By January 15, 2021, greater than 107,000 COVID-19 cases and 1,645 COVID-19-related deaths were reported in Libya. The Libyan government initiated lockdown measures and restrictive policies in March 2020; therefore, after 2 months of lockdown, there was a crucial need to address the mental health status of the Libyan population and to assess the socioeconomic status of people during the lockdown. Herein, we aim to provide an overview of the psychological status and behavioral consequences of the lockdown during COVID-19 in Libya.

## Materials and Methods

An online cross-sectional survey was conducted using Google Forms among the Libyan population through May and June 2020 in more than 20 cities. The survey was conducted anonymously using email and social media without identified data. The survey comprised basic demographic data of the participants and anxiety symptoms, measured using the seven-item Generalized Anxiety Disorder scale (GAD-7) (Spitzer et al., 2006) with ≥15 as the cut-off score for anxiety symptoms. The GAD-7 cut-off score of 15 or more was calculated by Gillis et al. (1995). The GAD-7 demonstrated good internal consistency, as determined by a Cronbach’s alpha level of 0.88 in a pilot study among the Libyan population. Additionally, a survey regarding the effect of lockdown was administered. The survey consisted of several parts, as follows: the first part involved the lockdown status, which measured the hours of lockdown, availability of services, number of working hours, and prevalence of abuse episodes; the second part involved lockdown activities, frequency of visitors, and shopping status before and during lockdown; the third part included internet activity and use during and before lockdown; the fourth part included sleep and weight change questions; and the fifth part included general health-related questions and the civil war effect on the Libyan population. The Libyan population numbered approximately 6,878,310 as of 30 July 2020 based on the Worldometer elaboration of the latest United Nations data (Worldometer, 2020). We estimated the required sample size based on a population size of 6,878,310 with a percentage estimated completion rate of 50% and a 95% confidence interval of 1.1 and a sample size of 7928 needed for the study.

### Ethical Considerations

Ethical approval for this study was obtained from the Bioethics Committee at the Biotechnology Research Center in Libya. All participants provided written informed consent before participating in the study.

### Statistical Analysis

Descriptive statistics were used to describe the data and survey responses using frequencies and percentages. A chi-square test was used to determine the association between anxiety symptoms and basic participant data characteristics. Mann–Whitney tests were used for continuous variables to compare differences. A Wilcoxon signed-rank test was used to determine the effect of lockdown on the number of people who visited the participants’ homes in the past week, changes in internet use, sleep time, and the time spent awake by estimating the pre- and post-effect of lockdown on these variables based on self-reports (each question contain choices that were put into a four-point scale to be used for estimating differences from pre- to post-lockdown on these variables and to determine if there is a median difference between two conditions). Binomial logistic regression was performed to ascertain the effects of study variables on the likelihood that participants have clinically significant anxiety symptoms (GAD ≥ 15). Data entry and analysis were performed using SPSS (version 25.0; IBM Corp., Armonk, NY, United States).

## Results

A total of 8084 responses were collected through the online survey. Of the participants, 5090 (63%) were women and 2994 (37%) were men. The mean age (SD) for study participants was 27.2 (8.9) years. Due to the online structure, we were not able to estimate the response rate. Table 1 shows an overview of the basic study characteristics and the associations of the cut-off score of anxiety symptoms (GAD score ≥ 15) with a high likelihood of anxiety symptoms; those with GAD < 15 were less likely to exhibit anxiety symptoms. Among the participants, 1145 (14.2%) reached the cut-off score to detect clinically significant anxiety symptoms; however, only gender, age range, marital status, educational level, job status, working status during the COVID-19 pandemic, number of hours worked per week, and being personally infected with COVID-19 were statistically associated with anxiety symptoms (*p* < 0.05). On the other hand, being the financial supporter of the family and having a friend or family member infected with COVID-19 were not associated with anxiety symptoms (*p* > 0.05).

**TABLE 1:** Basic study characteristics (*n* = 8084).

			Anxiety symptoms		
	*n* (%)	GAD ≥ 15	GAD < 15	χ^2^	P-value
Variables		*n* = 1145 (14.2%)	*n* = 6939 (85.8%)		
**Gender**				4.486	0.034*
Male	2994 (37)	392 (34.2)	2602 (37.5)		
Female	5090 (63)	753 (65.8)	4337 (62.5)		
Age, median (IQR) in years	25 (20–32)	23 (20–30)	25 (21–32)		<0.001**
**Age range (years)**				35.85	<0.001**
<25	3897 (48.2)	637 (55.6)	3260 (47)		
25–50	3999 (49.5)	497 (43.4)	3520 (50.5)		
>50	188 (2.3)	11 (1)	177 (2.6)		
**Marital status**				27.33	<0.001**
Married	2377 (29.4)	262 (22.9)	2115 (30.5)		
Not married (single, divorced, widowed)	5707 (70.6)	883 (77.1)	4824 (69.5)		
**Education level**				16.88	0.005*
Elementary	98 (1.2)	8 (0.7)	90 (1.3)		
Preparatory	431 (5.3)	53 (4.6)	378 (5.4)		
Secondary	2446 (30.3)	389 (34)	2057 (29.6)		
University education/higher education	4465 (55.2)	622 (54.3)	3843 (55.4)		
Postgraduate studies	550 (6.8)	67 (5.9)	483 (7)		
Other	94 (1.2)	6 (0.5)	88 (1.3)		
**Job status**				25.88	<0.001**
Full-time employee	2079 (25.7)	243 (21.2)	1836 (26.5)		
Freelance	1026 (12.7)	143 (12.5)	883 (12.7)		
Student	2845 (35.2)	438 (38.3)	2407 (34.7)		
Retired	46 (0.6)	2 (0.2)	44 (0.6)		
Unemployed	1657 (20.5)	269 (23.5)	1388 (20)		
Other	431 (5.3)	50 (4.4)	381 (5.5)		
**Work status during COVID-19 pandemic**				34.787	<0.001**
Still working	1639 (20.3)	167 (15.4)	1463 (21.1)		
Tele-working	939 (11.6)	103 (9)	836 (12)		
Suspended from work	5506 (68.1)	866 (75.6)	4640 (66.9)		
**Number of working hours per week**				23.93	<0.001**
Less than 30 h per week	3265 (40.4)	427 (37.3)	2838 (40.9)		
30–45 h per week	1552 (19.2)	183 (16)	1369 (19.7)		
More than 45 h per week	826 (10.2)	141 (12.3)	685 (9.9)		
Not applicable	2441 (30.2)	394 (34.4)	2047 (29.5)		
**Financial support for the family (yes) COVID-19 infection status**	1822 (22.5)	266 (23.2)	1556 (22.4)	0.37	0.545
Personally infected with COVID-19	93 (1.2)	2 (0.2)	91 (1.3)	11.17	<0.001**
A family member/relative infected	227 (2.8)	37 (3.2)	190 (2.7)	0.87	0.349
A friend/acquaintance	415 (5.1)	67 (5.9)	348 (5)	1.41	0.235

*Significant at p < 0.05; **Significant at p < 0.001.

A binomial logistic regression was performed to ascertain the effects of study variables on the likelihood of participants having clinically significant anxiety symptoms. The logistic regression model was statistically significant, χ^2^(22) = 165.67, *p* < 0.001. Of the study variables, only five were statistically significant predictors: age, gender, marital status, work status, being the financial supporter for the family, and being infected with COVID-19. Women had 1.19 times higher odds to exhibit anxiety symptoms than men. Increasing age was significantly associated with reduced likelihood of exhibiting anxiety symptoms, and being married was significantly associated with higher likelihood of anxiety symptoms, compared to not being married. Being suspended from work was associated with an increase in the likelihood of anxiety symptoms. However, we found that being infected with COVID-19 was associated with 9.59 times higher risk of exhibiting clinically significant anxiety symptoms. Table 2 illustrates an overview of the binominal logistic regression analysis.

**TABLE 2:** Binary logistic regression analysis of severe anxiety stress symptoms (GAD-7 ≥ 15).

Variables	n (%)	Odds ration	95% CI for odds ration		*p*-value
			Lower	Upper	
**Gender**					
Male	2994 (37)	1.00 (ref)			
Female	5090 (63)	1.19	1.04	1.37	0.01*
**Age range (years) mean (SD)/median (IQR)**	25 (8.9)/25 (20–32)	0.97	0.96	0.98	<0.001**
**Marital status**					
Married	2377 (29.4)	1.34	1.12	1.59	0.001*
Not married (single, divorced, widowed)	5707 (70.6)	1.00 (ref)			
**Education level**					
Elementary	98 (1.2)	1.00 (ref)			
Preparatory	431 (5.3)	1.16	0.38	3.55	0.78
Secondary	2446 (30.3)	1.47	0.6	3.57	0.4
University education/higher education	4465 (55.2)	2.11	0.9	4.93	0.08
Postgraduate studies	550 (6.8)	2.19	0.94	5.12	0.07
Other	94 (1.2)	2.25	0.93	5.44	0.07
**Job status**					
Full-time employee	2079 (25.7)	1.31	0.94	1.82	0.11
Freelance	1026 (12.7)	1.14	0.81	1.59	0.45
Student	2845 (35.2)	1.08	0.76	1.55	0.67
Retired	46 (0.6)	1.04	0.75	1.45	0.82
Unemployed	1657 (20.5)	1.00 (ref)			
Other	431 (5.3)	0.33	0.11	2.09	0.33
**Work status during COVID-19 pandemic**					
Still working	1639 (20.3)	1.07	0.82	1.39	0.62
Tele-working	939 (11.6)	1.00 (ref)			
Suspended from work	5506 (68.1)	1.57	1.3	1.89	<0.001**
**Number of working hours per week**					
Less than 30 h per week	3265 (40.4)	1.00 (ref)			
30–45 h per week	1552 (19.2)	0.87	0.74	1.03	0.1
More than 45 h per week	826 (10.2)	0.85	0.68	1.07	0.17
Not applicable	2441 (30.2)	1.32	1.02	1.7	0.035*
**Financial support for the family (yes) COVID-19 infection status**	1822 (22.5)	0.68	0.57	0.81	<0.001**
Personally infected with COVID-19	93 (1.2)	9.59	2.28	40.25	0.002*
A family member/relative infected	227 (2.8)	0.62	0.39	0.98	0.04*
A friend/acquaintance	415 (5.1)	0.78	0.55	1.09	0.14

*Significant at p < 0.05; **Significant at p < 0.001.

Regarding the lockdown status, most participants (6122 [75.7%]) thought that the nationwide lockdown was a good idea. Greater than half of the participants (4692 [58%]) reported staying at home all day during the lockdown, and 7234 (89.5%) reported the availability of basic life needs. Just under half of the participants (3347 [41.4%]) reported that they shared a residence with four to six people. Only 4108 (50.8%) participants reported that children were involved in home-schooling. However, this represented most of the 57.5% of the respondents with children at home during the lockdown. Table 3 summarizes the participants’ answers to the lockdown status survey.

**TABLE 3:** Lockdown status survey (*n* = 8084).

Questions	Yes/*n* (%)	No
**1.1 Do you personally believe that a nationwide lockdown was generally a good idea?**	6122 (75.7)	1962 (24.3)
**1.2 How many hours did you spend in the lockdown per day?**		
Less than 8 h	977 (12.1)	
8–12 h	1076 (13.3)	
12–18 h	1339 (16.6)	
Almost 24 h	4692 (58)	
**1.3 Were basic life necessities available to you during the lockdown (food, water, and medicine)?**	7234 (89.5)	850 (10.5)
**1.4 During lockdown, how many people did you share your residence with?**		
Alone	240 (3)	
1–3 people	1531 (18.9)	
4–6 people	3347 (41.4)	
7–9 people	2175 (26.9)	
10 or more people	791 (9.8)	
**1.5 During the lockdown, did you have any children living with you at your place of residence?**	4651 (57.5)	3433 (42.5)
**1.6 Did you find it more difficult than usual to care for your children after the lockdown?**	3026 (37.4)	5058 (62.6)
**1.7 Were the children involved in any homeschooling program during confinement?**	4108 (50.8)	3976 (49.2)
**1.8 Did you suffer any form of abuse during the lockdown? (tick all that apply)**		
Physical and/or verbal aggression from family members	1451 (17.9)	
Physical and/or verbal aggression from someone outside the family	958 (11.9)	
Physical and/or verbal aggression from enforcers implementing the lockdown	641 (7.9)	
**1.9 How would you best describe your life before the lockdown?**		
Very unsatisfactory	426 (5.3)	
Unsatisfactory	340 (4.2)	
Average	2237 (27.7)	
Satisfying	2019 (25)	
Excellent	3062 (37.9)	
**1.10 How would you best describe your life during the lockdown?**		
Very unsatisfactory	1942 (24)	
Unsatisfactory	1450 (17.9)	
Average	2580 (31.9)	
Satisfying	943 (11.7)	
Excellent	1169 (14.5)	
**1.11 Did you visit your workplace during the lockdown?**	2111 (26.1)	5973 (73.9)
**1.12 Will you continue to receive full salary during the lockdown?**	3294 (40.7)	4790 (59.3)
**1.13 Do you feel your life was put on hold or that your work progress slowed down after the lockdown?**	5589 (69.1)	2495 (30.9)

Among the study participants, 1451 (17.9%) reported a physical and/or verbal abuse episode from family members, 958 (11.9%) reported abuse outside the family, and 641 (7.9%) reported abuse from enforcers during the lockdown. We found an association of gender with abuse types: women encountered higher physical and/or verbal abuse from family members than men (10.6% vs. 7.3%, *p* < 0.001). The same higher rate of physical and/or verbal abuse from someone outside the family was found in women compared to men (7.9% vs. 3.9%, *p* = 0.009). However, physical and/or verbal aggression from enforcers implementing the lockdown was higher among men compared to women (4.4% vs. 3.5%, *p* < 0.001). A Wilcoxon signed-rank test was used to determine the effect of lockdown on life satisfaction; there was a statistically significant median decrease in life satisfaction from pre-lockdown (3.86) to post-lockdown (2.75; *p* ≤ 0.001). Of study participants, 3294 (40.7%) reported continuity of their salary, while 4790 (59.3%) reported a lack of income or salary during lockdown. Moreover, 5589 (69.1%) reported that their life was put on hold or their work stopped after the lockdown.

Most participants felt bored during the lockdown. Only 1231 (15.2%) participants did volunteer work during the lockdown. Most participants’ activities reported during lockdown were as follows: internet: 7749 (95.9%); watching TV: 5989 (74.1%); reading books: 4508 (55.8%); and studying: 2458 (30.4%). Table 4 provides an overview of the participants’ lockdown activities during the COVID-19 pandemic.

**TABLE 4:** Lockdown activity (*n* = 8084).

Questions	Yes/*n* (%)	No
**1.1 How often did you find yourself feeling bored and having nothing to do during the lockdown?**		
Never	862 (10.7)	
Sometimes	636 (7.9)	
Usually	1882 (23.3)	
Often	1840 (22.8)	
Always	2864 (35.4)	
**1.2 Did you participate in any volunteer work during the lockdown?**	1231 (15.2)	6853 (84.8)
**1.3 Type of activity during the COVID-19 lockdown**		
Studying	2458 (30.4)	
Reading books	4508 (55.8)	
Watching TV	5989 (74.1)	
Surfing internet	7749 (95.9)	
Other activities	6373 (78.8)	
**1.4 How often did you have visitors at your house before the lockdown?**		
Less than once a week	2176 (26.9)	
Once a week	1769 (21.9)	
Two to three times a week	2564 (31.7)	
Almost everyday	1575 (19.5)	
**1.5 How often did you have visitors at your house after the lockdown?**		
Less than once a week	5376 (66.5)	
Once a week	1225 (15.2)	
Two to three times a week	1096 (13.6)	
Almost everyday	387 (4.8)	
**1.6 Which effect of the lockdown caused you distress? (tick all that apply)**		
Inability to work	4170 (51.6)	
Education/training disruption	5064 (62.6)	
Inability to go to worship places	5117 (63.3)	
Inability to participate in social gathering activities	6401 (79.2)	
Inability to seek medical care	3405 (42.1)	
Forced to stay indoor	6349 (78.5)	
**1.7 On average, how often did you go out shopping before the lockdown?**		
Rarely	1981 (24.5)	
Once a week	2154 (26.6)	
Two to three times a week	2614 (32.3)	
At least once a day	1335 (16.5)	
**1.8 On average, how often did you go out shopping during the lockdown?**		
Rarely	5252 (65)	
Once a week	1511 (18.7)	
Two to three times a week	813 (10.1)	
At least once a day	508 (6.3)	

A Wilcoxon signed-rank test was used to determine the effect of lockdown on the number of visitors. There was a statistically significant median decrease in the number of visitors from pre-lockdown (2.44) to post-lockdown (1.15; *p* ≤ 0.001). A Wilcoxon signed-rank test was used to determine if there was a difference in shopping times per week pre- and post-lockdown. There was a statistically significant median decrease in shopping activities from pre- (2.41) to post-lockdown (1.48; *p* ≤ 0.001). Thus, shopping activities changed during the lockdown.

With respect to internet activities during the lockdown, most participants reported a notable increase in internet use (5320 [65.8%]). The main use of the internet for most participants was recreational (7281 [90.1%]) and keeping in touch with other people (7339 [90.8%]). Only 2409 (29.8%) participants reported enrollment in online courses, whereas 4057 (50.2%) reported that they used the internet to spread information about the COVID-19 pandemic. We used a Wilcoxon signed-rank test to determine if there were differences in keeping in touch with others using the internet before and during lockdown, and found a statistically significant median increase in use of the internet for contacting people from pre- (3.57) to post-lockdown (3.82; *p* ≤ 0.001). Table 5 shows internet activity use among study participants.

**TABLE 5:** Internet activity during the lockdown (*n* = 8084).

Questions	Yes/*n* (%)	No
**1.1 How did your use of the internet change after the lockdown?**		
Notably decreased	313 (3.9)	
Decreased to some extent	210 (2.6)	
Unchanged	1074 (13.3)	
Increased to some extent	1167 (14.4)	
Notably increase	5320 (65.8)	
**1.2 Main use of the internet during the lockdown? (tick all that apply)**		
Work	2676 (33.1)	
Recreational	7281 (90.1)	
Contacting other people	7339 (90.8)	
Educational	5619 (69.5)	
Other purposes	5997 (74.2)	
**1.3 Did you participate in any online courses during the COVID pandemic?**	2409 (29.8)	5675 (70.2)
**1.4 Did you use social media to spread any information about the COVID pandemic?**	4057 (50.2)	4027 (49.8)
**1.5 How often did you contact people using the internet before the lockdown?**		
Very rarely	799 (9.9)	
About once fortnightly	827 (10.2)	
Once a week	2383 (29.5)	
Two to three times a week	1119 (13.8)	
At least once a day	2956 (36.6)	
**1.6 How often did you contact people using the internet during the lockdown?**		
Very rarely	815 (10.1)	
About once fortnightly	516 (6.4)	
Once a week	1732 (21.4)	
Two to three times a week	1246 (15.4)	
At least once a day	3775 (46.7)	

We performed a Wilcoxon signed-rank test to determine if there were differences in the time of going to bed before and during the lockdown. There was a statistically significant median increase in the time going to bed being late from pre-lockdown (3.6) to post-lockdown (4.4), *p* < 0.001, which means that people tended to go to bed later at night during the lockdown, compared to pre-lockdown. Moreover, there was a statistically significant median increase in the time of waking up from pre- (3.6) to post-lockdown (4.7; *p* < 0.001), which means that participants woke up later during the lockdown period compared to pre-lockdown. Further, 4736 (58.6%) reported weight increase during lockdown. There was a statistically significant median decrease in performing physical exercise from pre- (1.83) to post-lockdown (1.65; *p* < 0.001), which means that people were engaging in less exercise during the lockdown period. Table 6 provides an overview of the sleep and weight changes.

**TABLE 6:** Sleep and weight changes (*n* = 8084).

Questions	Frequency (%)
**1.1 When did you usually go to bed before the lockdown?**	
9 pm or before	325 (4)
10 pm	1114 (13.8)
11 pm	1839 (22.7)
12 pm	3197 (39.5)
1 am	748 (9.3)
After 1 am	861 (10.7)
**1.2 When did you usually go to bed during the lockdown?**	
9 pm or before	170 (2.1)
10 pm	340 (4.2)
11 pm	645 (8)
12 pm	2714 (33.6)
1 am	2985 (36.9)
After 1 am	1230 (15.2)
**1.3 When did you usually wake up before the lockdown?**	
5 am or before	591 (7.3)
6 am	1319 (16.3)
7 am	1870 (23.1)
8 am	2207 (27.3)
9 am	969 (12)
After 9 am	1128 (14)
**1.3 When did you usually wake up during the lockdown?**	
5 am or before	479 (5.9)
6 am	325 (4)
7 am	530 (6.6)
8 am	2069 (25.6)
9 am	880 (10.9)
After 9 am	3801 (47)
**1.4 How did your body weight change after the lockdown?**	
No change	3348 (41.4)
Yes, increased	4736 (58.6)
**1.5 On average, how often did you perform physical exercise before the lockdown?**	
Rarely	4471 (55.3)
Once a week	1360 (16.8)
Two to three times a week	1430 (17.7)
More than three times a week	823 (10.2)
**1.6 On average, how often did you perform physical exercise during the lockdown?**	
Rarely	5331 (65.9)
Once a week	978 (12.1)
Two to three times a week	1008 (12.5)
More than three times a week	767 (9.5)

With respect to the general health status and civil war effect on the study population during the COVID-19 pandemic, we found that most of the participants (5726 [70.8%]) felt irritable, 5666 (70.1%) felt angry, 5178 (64.1%) reported difficulty in sleeping, and 5089 (64%) reported feeling sad. Table 7 provides an overview of the health and civil war status during the lockdown. Approximately one-half of the participants (2953 [36.5%]) reported obstacles to seeking medical care. Most participants reported that the civil war affected their quality of life to different degrees; specifically, 3782 (46.8%) experienced a devastating effect, 1120 (13.9%) had a significant effect, 1631 (20.2%) had a moderate effect, 497 (6.1%) minimum effect, and 1054 (13%) reported no effect. Approximately half of the participants (3480 [43%]) were targeted by attacks during the conflict, 2637 (32.6%) reported the death of a loved one or relative by the civil war, and 1812 (22.4%) were forced to relocate from their previous residence due to the civil war.

**TABLE 7:** General health and civil war status during the lockdown (*n* = 8084).

Questions	Yes/*n* (%)	No
**1.1 After the lockdown, did you experience any of the following issues more frequently than usual?**		
Headache/Migraine	4622 (57.2)	
Difficulty Sleeping	5178 (64.1)	
Difficulty Concentrating	4251 (52.6)	
Feeling Angry	5666 (70.1)	
Feeling Sad	5089 (63)	
Feeling fatigued	4972 (61.5)	
Muscle pain	4302 (53.2)	
Feeling irritable	5726 (70.8)	
**1.2 Did you face any difficulties accessing medical care?**	2953 (36.5)	5131 (63.5)
**1.3 Did you miss a doctor’s appointment or postpone a scheduled medical procedure due to the lockdown?**	3822 (47.3)	4262 (52.7)
**1.4 How did the recent civil war affect your quality of life?**		
Almost no effect	1054 (13)	
Minimum effect	497 (6.1)	
Moderate effect	1631 (20.2)	
Significant effect	1120 (13.9)	
Devastating effect	3782 (46.8)	
**1.5 Has your area or neighborhood been targeted by attacks during the recent civil war?**	3480 (43)	4604 (57)
**1.6 Did you suffer the death of a loved one or relative in the recent civil war?**	2637 (32.6)	5447 (67.4)
**1.7 Have you been forced to relocate from your previous residence due to the recent civil war?**	1812 (22.4)	6272 (77.6)

## Discussion

Our study focused on the Libyan population between May and June 2020, subsequent to the lockdown measures in March 2020. We measured the psychological and lockdown status of Libya during the COVID-19 pandemic.

Our study demonstrated several factors associated with anxiety symptoms, changes in behavior, lockdown activity status, internet activities, health status, civil war effects, and sleep and weight changes after commencing the lockdown. We demonstrated that 1145 (14.2%) of the study population reached the cut-off score to demonstrate clinically significant anxiety symptoms using the GAD-7 scale. Anxiety symptoms were statistically associated with gender, age, working status during the COVID-19 pandemic, being the family’s financial supporter, and being personally infected with COVID-19.

A previous study that was conducted in China during the early weeks of the COVID-19 pandemic found a prevalence of anxiety of 22.6% by using a GAD-7 cut-off score of ≥10 for identifying cases of anxiety symptoms (Gao et al., 2020). However, our study used a GAD-7 cut-off score of ≥15. Cao et al. (2020) conducted a study among 2279 participants from China during the COVID-19 pandemic using GAD-7 found that severe anxiety (GAD-7 > 15) was found in 0.9%, and moderate anxiety (GAD-7 10-14) in 2.7% of the study participants, which is lower than our results.

Approximately half of our participants (41.4%) reported that they shared residency with four to six other people, which raised concerns about the risk of spreading infection among the residents during lockdown. Another concern based on our findings was the rate of verbal and/or physical abuse, which is of concern because 17.9% of the participants experienced abuse from family members, 11.9% from someone outside their family, and 7.9% from law enforcers, during the lockdown. Women and children may have been at higher risk of domestic violence. According to UN Women (United-Nation, 2020; Vora et al., 2020), domestic violence increased substantially during 2020. Therefore, there is a need to implement regulations and policy to protect women and/or children who may be at risk of domestic violence. Our study was the first to demonstrate these rates in Libya, which can be explained by the financial instability due to the COVID-19 pandemic and the civil war effect. Indeed, the COVID-19 pandemic and civil war had catastrophic effects on the mental health status of the Libyan population (Vora et al., 2020).

There was a significant decrease in the life satisfaction of the study participants from before to during the lockdown. This is a major concern due to the lockdown effect, financial stress, and socioeconomic instability, which may need further assessment and urgent intervention. These concerns that happened during lockdown should alarm and inform the authorities to take actions and efforts to mitigate the negative consequence of COVID-19 pandemic on life satisfaction and well-being of people (Zhang et al., 2020).

Only 40.7% of the participants continued to receive their monthly salary during the lockdown and our study demonstrated that greater than 20.5% of the study population were unemployed. These findings are of significant importance because more than 69.1% of the participants reported that their workplace experienced difficulties due to the COVID-19 pandemic. The global impact of the COVID-19 pandemic increased the loans required by people, which was a financial catastrophe for many workers and employees who lost their jobs (Nicola et al., 2020). UNESCO estimated that approximately 900 million learners have been affected due to the disruption in education due to COVID-19 (Viner et al., 2020). Therefore, there is a need for an emergency relief plan for the Libyan population who are suffering from the COVID-19 pandemic on top of the adverse impact of the civil war (Elhadi and Msherghi, 2020). The authorities need to implement specific steps to alleviate the results of the pandemic on the socioeconomic lives of the Libyan population by providing loans, supporting online educational learning, increasing safety measures in public buildings and institutes, and providing major steps to facilitate the basic life supplies for people who are in need during difficult times.

Variety of activities were reported during the lockdown: most participants reported internet surfing (95.9%) and watching TV (74.1%). We also observed a significant decrease in terms of visits to meet others per week, before and after the lockdown. This means that people are likely to adhere to the social distancing rules; however, there were still some participants reporting visits by others two to three times per week (13.6%) and nearly every day (4.8%). Additionally, we observed a significant decrease in shopping times per week compared with the pre-lockdown period, although there were still some participants reporting shopping everyday (6.3%) and two to three times per week (10.1%). Therefore, there is a need to implement more guidance for people to prevent the risk of transmission. There is also a need to implement online shopping because of the lack in Libya, which can reduce this burden and let the stores deliver the daily needs of people at home to reduce the risk of gathering and therefore reduce the risk of transmission (Sheth, 2020).

We observed a significant increase in internet use during the lockdown by comparing it to the previous period before the lockdown; however, this increase in internet use can have negative consequences, because there is a concern of an increased risk of gambling, video gaming, and watching inappropriate content, which may have negative effects on society, as they are being used to reduce the stress-related effects of the lockdown. Therefore, there is a need for further discussion and measuring the prevalence and effects of these behaviors among people in the lockdown (Király et al., 2020). Moreover, another observation in our study was that 50.2% of the participants reported using the internet to spread information related to the COVID-19 pandemic, which can question the reliability of the information and whether false or misleading health-related information is being spread (Cuan-Baltazar et al., 2020).

We observed sleep changes during the COVID-19 lockdown. Specifically, people reported going to bed later during the lockdown compared to the previous period. We also observed a significant increase in the wake-up hours compared to the period prior to the lockdown. Therefore, there is a high likelihood of insomnia and sleep disturbances that need to be addressed in future, as insomnia and sleep disturbances have been reported during the COVID-19 pandemic (Morin and Carrier, 2020).

Among the study participants, 58.6% reported an increase in body weight. Additionally, we found a significant decrease in physical exercise by comparing the frequency of exercise before and during the lockdown. Both, an increase in weight and a reduction in physical exercise, were reported by the study participants. These behavioral changes carry a higher risk of obesity and metabolic syndrome and increase the risk of severe COVID-19 infection (Sanchis-Gomar et al., 2020).

Most participants also felt irritable (70.8%), angry (70.1%), and fatigued (61.5%). These behavioral changes are of major importance; the increase in risk of psychological stress-related issues, and the increase in prevalence of depression, anxiety, and post-traumatic stress disorders among study participants need to be addressed. Moreover, we found that one-third of the participants had difficulty in accessing healthcare services, which may increase the risk of chronic disease deterioration (Liu et al., 2020).

Most participants reported that the civil war affected their quality of life. The civil war had great psychological and socioeconomic effects on the quality of life of people and increased their stress due to fear and uncertainty. In addition, 43% of the participants were targeted by the conflict, 32.6% reported that they suffered a death of a loved one, and 22.4% were internally displaced due to the civil war. These negative effects had catastrophic consequences on the Libyan population and there is a need for longitudinal studies that provide more detailed information about the current status of the Libyan population during the civil war. Further, there is a need for actions to alleviate the difficulties and challenges endured by the Libyan people and to find socioeconomic and psychological intervention solutions to provide relief for the Libyan population.

### Strength and Limitations

This study demonstrated a high level of stress among Libyans during the COVID-19 pandemic lockdown, with some more susceptible than others to experiencing clinically significant anxiety during the lockdown. The study was conducted using a large sample of 8084 participants with wide demographic and socioeconomic variations. There was a significant decrease in life satisfaction from pre- to during-lockdown and several important behavioral and psychological changes during the lockdown were observed.

Our study was a cross-sectional study, which may not have the ability to draw strong conclusions regarding causation. Another limitation that needs to be acknowledged is that the cross-sectional nature of the survey meant that changes from pre- to during-lockdown in the outcomes had to be assessed retrospectively, and participants may have recall bias, which may have affected the accuracy of their retrospective memory to these questions. Thus, future longitudinal studies to support or dispute our results are warranted, especially in the elderly population, who are known to have a higher risk of depressive and anxiety symptoms, compared to the younger population. Another limitation was that the study used an online survey, and thus, data for people without an internet connection during the study period was not collected. Additionally, the civil war effect was not addressed in detail and further studies are needed to address the post-traumatic stress disorder and depressive symptoms to measure the prevalence of possible stressful negative consequences of the civil war in further detail. Further, because we introduced a new tool for measuring the lockdown status, there is a need for further studies to validate the psychometric properties of our tool and to use it in different settings with different countries.

## Conclusion

Our study provided an overview of the psychological and behavioral status among those who resided in Libya during the civil war and COVID-19 pandemic. The study demonstrates a concerningly high level of clinically significant anxiety during lockdown among the Libyan population during Libya’s lockdown period. There is a need to address and solve the negative consequences of the lockdown to prevent further deterioration of the Libyan people’s mental and socioeconomic status.

## Data Availability Statement

The raw data supporting the conclusions of this article will be made available by the authors, without undue reservation.

## Ethics Statement

The studies involving human participants were reviewed and approved by the Biotechnology Research Center in Libya. The patients/participants provided their written informed consent to participate in this study.

## Author Contributions

AhA, AlA, and ME conceptualized the idea. AM and ME analyzed the data, revised the manuscript in response to reviewer comments and revised the manuscript for language and clarity and grammar. AM revised the statistics with ME and helped in preparing the results and tables. ME wrote the initial draft of the manuscript. Each author contributed to data collections, participated in revising the manuscript, and all agreed to accept equal responsibility for this manuscript’s accuracy. All authors approved the final manuscript.

## Conflict of Interest

The authors declare that the research was conducted in the absence of any commercial or financial relationships that could be construed as a potential conflict of interest.
